# Comparison of mortality and cardiovascular complications due to COVID-19, RSV, and influenza in hospitalized children and young adults

**DOI:** 10.1186/s12872-024-04366-0

**Published:** 2024-11-28

**Authors:** Sagya Khanal, Bishes Khanal, Fu-Sheng Chou, Anita J. Moon-Grady, Laxmi V. Ghimire

**Affiliations:** 1 Nepal Medical College and Teaching Hospital, Kathmandu, Nepal; 2 Kathmandu Medical College and Teaching Hospital, Sinamangal, Kathmandu, Nepal; 3 Department of Neonatal-Perinatal Medicine, Kaiser Permanente Riverside Medical Center, Riverside, CA USA; 4 Division of Pediatric Cardiology, UCSF Benioff Children’s Hospital, Department of Pediatrics, University of California, San Francisco, San Francisco, CA USA; 5 Division of Pediatric Cardiology, Fresno Regional campus, University of California, CA San Francisco, USA

**Keywords:** COVID-19, Cardiovascular complications, Hospital outcomes, Influenza, Mortality, RSV

## Abstract

**Background:**

Respiratory viruses are linked to cardiovascular complications. We aim to compare cardiovascular complications due to COVID-19, influenza and RSV.

**Methods:**

We analyzed cross-sectional data from hospitalized children and young adults (≤ 20 years) from 2020 and 2021 using National Inpatient Sample (NIS). We included individuals hospitalized for COVID-19, RSV, and influenza, and weighted data were used to compare cardiovascular complications.

**Results:**

Of 212,655 respiratory virus admissions, 85,055 were from COVID-19, 103,185 were from RSV, and 24,415 were from influenza. Myocarditis was higher in COVID-19 [0.9%, *n* = 740] as compared to influenza [0.2%, *n* = 55] and RSV [0.1%, *n* = 65]. In the adjusted logistic regression, the odds of myocarditis was 61% lower in influenza [aOR = 0.39 (0.20–0.76), *P* = 0.006], and 85% lower in RSV [aOR = 0.15 (0.07–0.34) *P* < 0.001] as compared to COVID-19. Bradyarrhythmias/heart block was higher in COVID-19 [0.8%, *n* = 690] versus influenza [0.5%, *n* = 110] and RSV [0.2%, *n* = 205]. After adjusting for confounders for bradyarrhythmias/heart block, compared to COVID-19, the odds were 49% lower in RSV [aOR = 0.51 (0.33–0.80), *P* = 0.004] but no statistically significant difference in influenza [aOR = 0.79 (0.48–1.31), *P* = 0.374] was seen. Tachyarrhythmias, sudden cardiac arrest, and in-hospital mortality showed no differences after adjusting for covariates.

**Conclusion:**

Individuals with COVID-19 infection are more likely to develop cardiovascular complications compared to influenza and RSV, highlighting the need for higher index of suspicion and prompt treatment, as well as steps to limit infection and transmission of this virus in children.

**Supplementary Information:**

The online version contains supplementary material available at 10.1186/s12872-024-04366-0.

## Background

Respiratory viral infections represent a significant public health challenge, contributing substantially to both morbidity and mortality [1]. Common respiratory viral pathogens include adenovirus, enterovirus, human coronavirus, human metapneumovirus, rhinovirus (RV), influenza, parainfluenza, and respiratory syncytial virus (RSV) [2]. Notably, COVID-19, influenza, and RSV exhibit similar clinical presentations and share common transmission routes through droplets and aerosols, which complicates their clinical differentiation [1].

Respiratory syncytial virus (RSV) is the leading cause of hospital admissions in infants and young children, [3] while influenza and SARS-CoV-2 are more prevalent in older children [4]. Previous studies have established a connection between respiratory viral infections and major cardiovascular complications [5–18]. RSV infection has been associated with myocarditis, [5] ventricular tachycardia, [6, 7] and heart block [8, 9] Influenza infection is a recognized cause of myopericarditis [10–12] and is associated with an elevated risk of acute heart failure and acute ischemic heart disease [13]. SARS-CoV-2 infection manifests a wide range of clinical presentations, including myocardial involvement such as myocarditis, dysrhythmias, heart failure, myocardial infarction, and thromboembolic events [14–18].

The emergence of SARS-CoV-2 in 2019 led to a significant increase in hospitalizations across all age groups and was associated with a high risk of in-hospital mortality [19]. Although cardiovascular manifestations of respiratory viral infections have been documented, [5, 6, 8, 10, 13, 14, 18] they have largely been reported separately. To our knowledge, no study has systematically compared the clinical complications and outcomes associated with COVID-19, influenza, and RSV infections in the pediatric population. This study aims to compare in-hospital mortality and major cardiovascular complications among hospitalized children and young adults with COVID-19, influenza, and RSV infections.

## Methods

### Study population and variables

This retrospective study utilized hospital discharge records from the National Inpatient Sample (NIS) for the years 2020 and 2021. The NIS is a component of the Healthcare Cost and Utilization Project (HCUP), funded by the Agency for Healthcare Research and Quality (AHRQ) [20]. The NIS sampling frame encompasses data from 48 statewide data organizations, including 47 states plus the District of Columbia, representing approximately 98% of the U.S. population. It includes a stratified 20% sample of discharges from U.S. community hospitals, excluding long-term acute care hospitals and rehabilitation facilities. The NIS ensures patient confidentiality because of the de-identified nature of data. A detailed description of the database is available on the HCUP website [20].

**Fig. 1:**
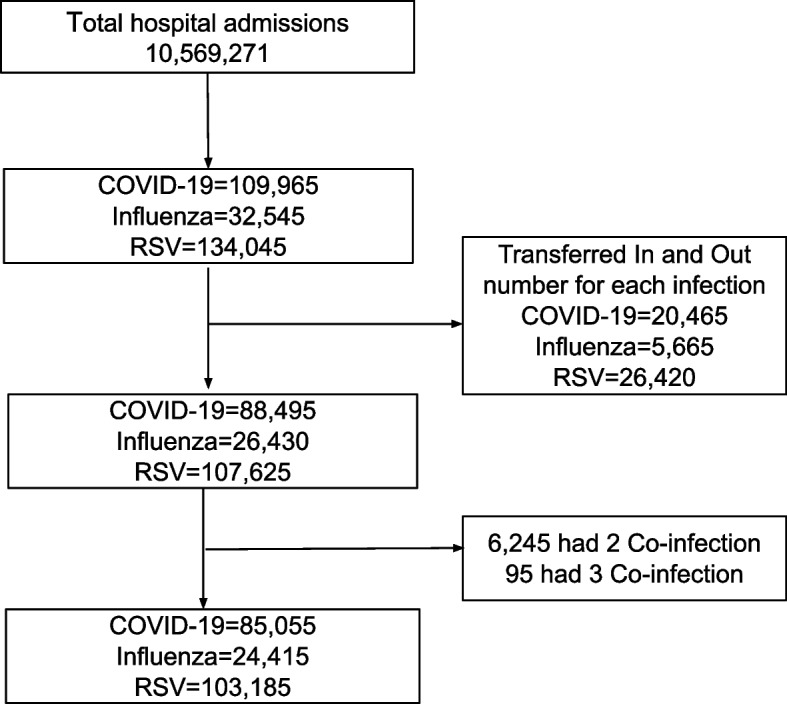
Flow diagram for in-hospital admission for COVID-19, influenza and RSV infection

Our study focused on individuals aged 20 years or younger who were hospitalized with a diagnosis of COVID-19, influenza, or respiratory syncytial virus (RSV). The International Classification of Diseases, Tenth Revision, Clinical Modification (ICD-10-CM), was utilized to identify hospitalized children diagnosed with COVID-19, influenza, RSV, and other variables studied. To minimize confounding effects, we excluded individuals with co-respiratory infections (i.e., those diagnosed with more than one of the studied viral infections: COVID-19, influenza, or RSV) and those who were transferred between hospitals to prevent double counting (Fig. 1).

To identify COVID-19 cases within the database, we utilized the ICD-10-CM code B97.29 for records from January 1 to March 31, 2020, and U07.1 for records from April 1, 2020, onward [21]. For RSV infection, the following ICD-10-CM codes were employed: B97.4, J12.1, J20.5, and J21.0. We excluded cases of bronchiolitis that did not have a corresponding RSV diagnosis. We excluded admissions that did not have complete data for analysis.

Severity, used as a variable in the analysis, denotes the severity of illness and has four categories: 1) minor loss of function, which includes cases with no comorbidity or complications; 2) moderate loss of function; 3) major loss of function; and 4) extreme loss of function [22].

### Outcome variables

The primary outcomes were in-hospital mortality and major cardiovascular complications associated with COVID-19, influenza, or RSV infection. For the purpose of analysis, major cardiovascular complications were myocarditis, tachyarrhythmia, bradyarrhythmias/heart block, sudden cardiac arrest, and the need for extracorporeal membrane oxygenation (ECMO). Additionally, we compared disease severity and length of hospital stay across the study groups. The ICD-10 codes corresponding to the diagnoses and procedures analyzed in this study are presented in supplementary Table 1.

### Statistical analysis

We conducted both descriptive and inferential analyses, followed by logistic regression modeling, to evaluate the data. Given that the NIS is a complex survey dataset, we incorporated clusters, strata, and weighting as recommended by the Healthcare Cost and Utilization Project (HCUP) to generate national estimates and ensure the accuracy of the statistical analyses. Continuous variables, such as age, length of stay, and disease severity, were reported as medians with interquartile ranges. Categorical variables were analyzed using weight-adjusted chi-square tests.

For multivariable analysis, we selected variables with reliable and consistent ICD-10 codes. Initially, univariable regression analysis was performed for variables of interest, followed by multivariable regression analysis that accounted for additional covariates, including age group, sex, asthma/reactive airway disease, prematurity, obesity, diabetes, congenital heart disease, chromosomal anomalies, ZIP code of household neighborhood, and disease severity. These covariates were identified through a comprehensive literature review and clinical expertise and were finalized prior to conducting the analyses.

The patient’s ZIP code was categorized into quartiles based on the estimated median household income of residents within that ZIP code, with quartiles representing the range from the lowest to highest income, indicating the poorest to wealthiest populations. All statistical analyses were conducted using Stata statistical software (version 15.1) and R (version 4.3) with RStudio (version 1.2). Figures were generated using the ggplot2 package in R [23].

## Results

Of 212,655 respiratory virus admissions, 85,055 were from COVID-19, 24,415 were from influenza, and 103,185 were from RSV. Among these, 46,845 (55.1%) of the COVID-19 cases, 11,290 (46.3%) of the influenza cases, and 46,445 (45.0%) of the RSV cases were female patients. The median age of children hospitalized with COVID-19 was 15 years (IQR: 3–19), with influenza it was 4 years (IQR: 1–9), and with RSV it was under 1 year (IQR: 0–1). COVID-19 admissions were more prevalent among young adults (11–20 years), whereas influenza and RSV admissions were more common among younger children (0–2 years).

Among children with underlying medical conditions, hospitalized children and young adults with a history of asthma/reactive airway disease were more common in those with influenza (21.6%, *n* = 5,265), followed by COVID-19 (14.4%, *n* = 12,260) and RSV (13.6%, *n* = 14,040). Children with obesity were more likely to have COVID-19 (14.2%, *n* = 12,095) compared to influenza (2.1%, *n* = 510) and RSV (0.5%, *n* = 485). Additionally, prematurely born children were more likely to have RSV (4.5%, *n* = 4,660) compared to COVID-19 (1.5%, *n* = 1,280) and influenza (2.1%, *n* = 505) (Table 1).

**Table 1:** Characteristics of patients, comorbid conditions, and complications due to COVID-19, influenza and RSV. Total Respi virus cases: 212,655

Variables	Respiratory virus with total number(percentages)			
	COVID-19	Influenza	RSV	*P* Value
Total number (n)	85,055	24,415	103,185	N/A
Female	46845 (55.1)	11290 (46.3)	46445 (45.0)	<0.001
Age, years (median [IQR])	15 [3-19]	4 [1-9]	0 [0-1]	<0.001
Age group (year)				<0.001
0-2	20405 (24.0)	10015 (41.0)	91700 (88.9)	
3-5	4455 (5.2)	4265 (17.5)	7820 (7.6)	
6-10	7235 (8.5)	4805 (19.7)	2150 (2.1)	
11-20	52960 (62.3)	5330 (21.8)	1515 (1.5)	
Zip Code^d^				<0.001
1st quartile	30980 (36.9)	8365 (34.6)	32290 (31.6)	
2nd quartile	21540 (25.6)	6095 (25.2)	26500 (25.9)	
3rd quartile	18870 (22.5)	5695 (23.5)	24180 (23.6)	
4th quartile	12655 (15.1)	4045 (16.7)	19340 (18.9)	
Comorbid conditions				
CHD^a^	2530 (3.0)	970 (4.0)	4985 (4.8)	<0.001
Obesity	12095 (14.2)	510 (2.1)	485 (0.5)	<0.001
Diabetes	5085 (6.0)	710.0 (2.9)	270 (0.3)	<0.001
Chromosomal Anomalies	2180 (2.6)	730 (3.0)	2250 (2.2)	0.004
Asthma/reactive airway	12260 (14.4)	5265 (21.6)	14040 (13.6)	<0.001
Prematurity	1280 (1.5)	505 (2.1)	4660 (4.5)	<0.001
Cardiovascular Complications				
Myocarditis	740 (0.9)	55 (0.2)	65 (0.1)	<0.001
Tachyarrhythmia	1290 (1.5)	235 (1.0)	635 (0.6)	<0.001
Supraventricular	525 (0.6)	100 (0.4)	355 (0.34)	<0.001
Ventricular	600 (0.7)	85 (0.4)	180 (0.2)	<0.001
Bradyarrythmia/Heart Block	690 (0.8)	115 (0.5)	205 (0.2)	<0.001
Sudden Cardiac Arrest	310(0.4)	50(0.2)	135 (0.1)	<0.001
ECMO^b^	170 (0.2)	55 (0.2)	45 (0.0)	<0.001
Length of stay (LOS) (Median[IQR]) in days	3 [2-5]	2 [2-4]	3 [2-4]	<0.001
Disease Severity (Median[IQR])^c^	3 [2-3]	2 [1-3]	2 [1-3]	<0.001
In-hospital mortality	580 (0.7)	65 (0.3)	130 (0.1)	<0.001

^a^Congenital Heart Disease

^b^Extracorporeal membrane oxygenation

^c^Severity illness subclass according to loss of function

^d^Zip Code: Neighborhood ZIP Codes classify the estimated median household income of residents in a patient's ZIP Code into four quartiles. The quartiles are identified from lowest to highest, indicating the lowest-income neighborhoods to highest-income neighborhoods

We performed univariable and multivariable logistic regression analyses to compare in-hospital mortality and major cardiovascular complications across COVID-19, influenza, and RSV cases. Multivariable logistic regression was performed after adjusting for confounding factors including age group, gender, prematurity, obesity, diabetes, asthma, congenital heart disease, chromosomal anomalies, and disease severity. Using COVID-19 as the reference group, we assessed the risk of complications associated with influenza and RSV.

The in-hospital mortality rate was 0.7% (*n* = 580) for COVID-19, 0.3% (*n* = 65) for influenza, and 0.1% (*n* = 130) for RSV. Descriptive analysis indicated higher in-hospital mortality for COVID-19 compared to influenza and RSV. However, when adjusted for covariates, the differences in in-hospital mortality were not statistically significant, with an adjusted odds ratio (aOR) of 0.92 (95% CI: 0.49–1.71, *P* = 0.799) for influenza and 0.67 (95% CI: 0.39–1.14, *P* = 0.142) for RSV, relative to COVID-19 (Table 2). In this model, those with diabetes and higher disease severity were associated with increased risk of in-hospital mortality. The descriptive statistics table with individuals who died vs those who survived are presented in Table 3.

**Table 2:** Logistic Regression of in-hospital mortality, cardiovascular and non-cardiovascular complications. (Taking COVID-19 as reference)

Complications	Respiratory viruses	Unadjusted Odds Ratio (95% CI)	*P* value	Adjusted Odds Ratio (95% CI)	*P* value
In- hospital Mortality	Reference (COVID-19)	-			
	Influenza	0.38 (0.21–0.69)	0.01	0.92 (0.49–1.71)	0.799
	RSV	0.18 (1.11–0.28)	0.00	0.67 (0.39–1.14)	0.142
Cardiovascular Complications					
Myocarditis	Reference (COVID-19)	-			
	Influenza	0.25 (0.14–0.47)	< 0.001	0.39 (0.20–0.76)	0.006
	RSV	0.049(0.02–0.09)	< 0.001	0.15 (0.07–0.34)	< 0.001
Bradyarrhythmia/Heart Block	Reference (COVID-19)	-			
	Influenza	0.57 (0.37–0.90)	0.015	0.79 (0.48–1.31)	0.374
	RSV	0.24 (0.17–0.34)	< 0.001	0.51 (0.33–0.80)	0.004
Tachyarrhythmia	Reference (COVID-19)	-			
	Influenza	0.63 (0.46–0.86)	0.004	1.21 (0.85–1.74)	0.277
	RSV	0.40 (0.32–0.49)	< 0.001	1.15 (0.84–1.59)	0.366
Supraventricular arrhythmia	Reference (COVID-19)	-			
	Influenza	0.7 (0.4–1.1)	0.092	1.2 (0.7–2.1)	0.49
	RSV	0.6 (0.4–0.8)	< 0.001	1.1 (0.7–1.8)	0.55
Ventricular arrhythmia	Reference (COVID-19)	-			
	Influenza	0.5 (0.3–0.8)	0.006	1.1 (0.6–1.9)	0.73
	RSV	0.2 (0.2–0.4)	< 0.001	1.1 (0.6–1.8)	0.80
Sudden Cardiac arrest	Reference (COVID-19)	-			
	Influenza	0.56 (0.28–1.09)	0.09	1.14 (0.55–2.35)	0.722
	RSV	0.35 (0.22–0.56)	< 0.001	0.85 (0.49–1.47)	0.569

**Table 3:** Stratification of respiratory virus cases and complications by those who died vs those who survived

Variables	Died (number and percentages)		*P*-value
	No	Yes	
Total number	211,829	775	N/A
Female	104,235 (49.2)	315 (40.6)	0.039
Age (median[IQR])	2 [0–12]	15 [2-19]	< 0.001
Age group (in years)			< 0.001
0–2	121,889 (57.54)	215 (27.74)	
3–5	16,504 (7.79)	30 (3.87)	
6–10	14,105 (6.66)	80 (10.32)	
11–20	59,329 (28.01)	450 (58.06)	
Zip Code^d^			0.073
1st quartile	71,320 (34)	290 (38.2)	
2nd quartile	53,945 (25.7)	185 (24.3)	
3rd quartile	48,525 (23.1)	210 (27.6)	
4th quartile	35,960 (17.1)	75 (9.9)	
Respiratory viruses			< 0.001
COVID-19	84,445 (39.9)	580 (74.8)	
Influenza	24,350 (11.5)	65 (8.4)	
RSV	103,035 (48.6)	130 (16.8)	
Comorbid conditions			
CHD^a^	8400 (4.0)	85 (11.0)	< 0.001
Obesity	12,950 (6.1)	140 (18.1)	< 0.001
Diabetes	5990 (2.8)	70 (9.0)	< 0.001
Chromosomal Anomalies	5115 (2.4)	45 (5.8)	0.006
Asthma	31,430 (14.8)	130 (6.8)	0.50
Prematurity	6425 (3.0)	20 (2.6)	0.75
Cardiovascular Complications			
Myocarditis	825 (0.4)	15 (1.9)	0.002
Tachyarrhythmia	2060 (1.0)	95 (12.3)	< 0.001
Bradyarrhythmia/Heart Block	995 (0.5)	15 (1.9)	< 0.001
Sudden Cardiac Arrest	255 (0.1)	235 (30.3)	< 0.001
ECMO^b^	165 (0.1)	125 (16.1)	< 0.001
In-hospital mortality	0 (0)	775 (100)	< 0.001

^a^Congenital Heart Disease

^b^Extracorporeal membrane oxygenation

^c^Severity illness subclass according to loss of function

^d^Zip Code: Neighborhood ZIP Codes classify the estimated median household income of residents in a patient's ZIP Code into four quartiles. The quartiles are identified from lowest to highest, indicating the lowest-income neighborhoods to highest-income neighborhoods

**Fig. 2:**
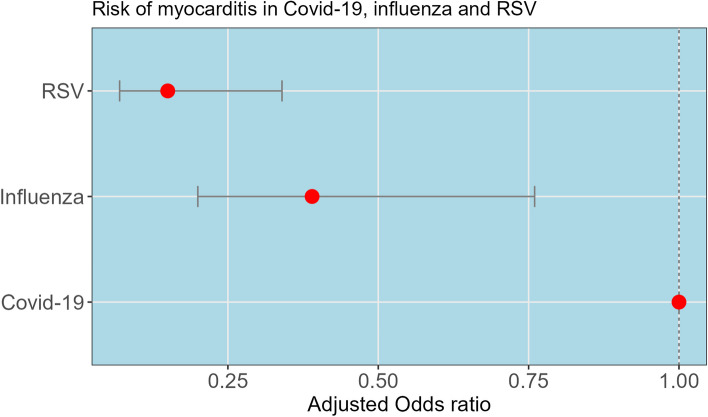
Risk of myocarditis in COVID-19, influenza and RSV infection

Regarding cardiovascular complications, myocarditis was more frequent in COVID-19 cases (0.9%, *n* = 740) compared to influenza (0.2%, *n* = 55) and RSV (0.1%, *n* = 65) cases in descriptive analyses. The risk of myocarditis was 61% lower in influenza with an adjusted odds ratio (aOR) of 0.39 (95% CI: 0.20–0.76, *P* = 0.006) and 85% lower in RSV with an adjusted odds ratio (aOR) of 0.15 (95% CI: 0.07–0.34, *P* < 0.001) compared to COVID-19 (Table 2 and Fig. 2). The descriptive statistics table with individuals with myocarditis vs those without myocarditis are presented in (Table 3). Similarly, the risk of bradyarrhythmia/heart block was 49% lower in RSV with an adjusted odds ratio (aOR) of 0.51 (95% CI: 0.33–0.80, *P* = 0.004) compared to COVID-19, though it was not statistically significant for influenza (aOR 0.79, 95% CI: 0.48–1.31, *P* = 0.374). While descriptive analyses suggested that tachyarrhythmias (including ventricular and supraventricular) and sudden cardiac arrest were more common in COVID-19, these findings were not statistically significant in the multivariable logistic regression models (Table 2).

The median length of hospital stay was 3 days (IQR: 2–5) for COVID-19, 2 days (IQR: 2–4) for influenza, and 3 days (IQR: 2–4) for RSV (Table 1).

## Discussion

Using the NIS 2020–2021 database, we report the following major findings. First, the in-hospital mortality rates were similar for COVID-19, influenza, and RSV infections requiring hospitalization. Second, the risk of cardiovascular complications, particularly myocarditis and bradyarrhythmia/heart block, were more common in COVID-19.

The similarity in in-hospital mortality rates among these respiratory viruses, after adjusting for confounders, aligns with findings from previous studies. For instance, a study conducted in Mexico by Laris-González et al. reported comparable in-hospital mortality in multivariable analysis between COVID-19 and influenza [24]. Hedberg et al., in their study based in Sweden, compared clinical phenotypes and outcomes of different respiratory viral infections in both pediatric (≤ 15 years) and adult cohorts. Their results indicated that in the adult cohort, 30-day mortality was significantly higher for COVID-19 compared to influenza (aHR 4.43, 95% CI: 3.51 to 5.59) and RSV (aHR 3.81, 95% CI: 2.72 to 5.34). However, in the pediatric cohort, the comparison of death rates (30-day and 90-day) was similar [25]. The milder course of COVID-19 in young children and infants, compared to adults, may partially explain the similar mortality rates observed for COVID-19, influenza, and RSV [26].

The risk of myocarditis was notably higher in COVID-19 compared to influenza and RSV. This was particularly evident in young adults aged 11–20 years, with obesity identified as a common comorbid condition (Table 4). The higher prevalence of myocarditis in COVID-19 may be attributed to the distinct pathophysiological mechanisms and immune responses triggered by SARS-CoV-2. As a cardiotropic virus, SARS-CoV-2 significantly impacts myocardial tissue and the cardiac conduction system, causing myocarditis by binding to ACE2 (Angiotensin-Converting Enzyme 2) receptors expressed in myocardial cells, pericytes, and pneumocytes, leading to direct cellular damage and uncontrolled inflammatory responses [27]. Additionally, endothelial dysfunction has been proposed to play an influential role in the pathophysiology of the SARS-CoV-2 infection [28]. Consistent with our findings, data from the Centers for Disease Control and Prevention (CDC) indicated that myocarditis was 16 times more prevalent in COVID-19 patients than in those without the infection, particularly among older children, younger adolescents (< 16 years), and older adults (> 75 years) [29]. In a subset analysis using NIS 2020 data, we previously compared clinical outcomes in myocarditis cases associated with COVID-19 versus non-COVID-19 cases and found that while the risk of mortality was similar between the two groups, acute kidney injury was more common in COVID-19-associated myocarditis (aOR = 1.9, 95% CI: 1.1–3.3, *P* = 0.02). In that study, rates of tachyarrhythmias, heart blocks, sudden cardiac arrest, and ECMO use were similar between the two groups [30].

**Table 4:** Stratification of respiratory virus cases and complications by those with and without myocarditis

Variables	Myocarditis (number and percentages)		*P*-value
	No	Yes	
Total number	211,815	840	N/A
Female	104,300 (49.2)	280 (33.33)	< 0.001
Age (median[IQR])	2 [0–12]	11 [6-17]	< 0.001
Age group (in years)			< 0.001
0–2	12,010 (57.6)	110 (13.1)	
3–5	16,470 (7.8)	70 (8.338)	
6–10	13,970 (6.6)	220 (26.2)	
11–20	59,365 (28.0)	440 (52.4)	
Zip Code^d^			0.92
1st quartile	71,355 (34.0)	280 (33.5)	
2nd quartile	53,925 (25.7)	210 (25.1)	
3rd quartile	48,560 (23.2)	185 (22.2)	
4th quartile	35,880 (17.1)	160 (19.2)	
Respiratory viruses			
COVID-19	84,315 (39.8)	740 (88.1)	< 0.001
Influenza	24,360 (11.5)	55 (6.5)	0.046
RSV	103,140(48.7)	45 (5.4)	< 0.001
Comorbid conditions			
CHD^a^	8455 (4.0)	30 (3.6)	< 0.001
Obesity	12,990 (6.1)	100 (11.9)	0.001
Diabetes	6050 (2.9)	15 (1.8)	0.40
Chromosomal Anomalies	5150 (2.439)	10 (1.2)	0.29
Asthma	31,430 (14.8)	135 (16.1)	0.67
Prematurity	6440 (3.04)	5 (0.6)	0.75
Cardiovascular complications			
Tachyarrhythmia	2055 (1.0)	105 (12.5)	< 0.001
Bradyarrhythmia/Heart Block	965 (0.5)	45 (5.4)	< 0.001
Sudden Cardiac Arrest	480 (0.23)	15 (1.8)	< 0.001
ECMO^b^	265 (0.13)	25 (3.0)	< 0.001
In-hospital mortality	760 (0.4)	15 (1.8)	0.002

^a^Congenital Heart Disease

^b^Extracorporeal membrane oxygenation

^c^Severity illness subclass according to loss of function

^d^Zip Code: Neighborhood ZIP Codes classify the estimated median household income of residents in a patient's ZIP Code into four quartiles. The quartiles are identified from lowest to highest, indicating the lowest-income neighborhoods to highest-income neighborhoods

Studies on the risk of bradyarrhythmia/heart block associated with various respiratory viral infections have been conducted separately. In this study, we compared the risk of bradyarrhythmia/heart block across COVID-19, influenza, and RSV. We found that it was more common in COVID-19 compared to RSV, although the difference was not statistically significant when compared to influenza. The exact mechanism by which COVID-19 leads to bradyarrhythmia/heart block remains unclear. Some studies suggest that heart block may result from direct disruption of the heart's electrical conduction system by the SARS-CoV-2 virus. It has been proposed that direct viral infiltration of cardiomyocytes via angiotensin-converting enzyme 2 (ACE2) receptors, followed by systemic inflammation, may contribute to cardiac injury [31]. During the 2009 influenza pandemic, Ukimura et al. reported four cases of influenza-related complete heart block (CHB) requiring temporary pacing, highlighting the potential for severe cardiac complications like heart block during respiratory viral infections [32]. Few studies seem to investigate and compare this complication due to these respiratory viruses. This gap in the literature highlights the necessity of further exploration and understanding of this specific complication of viral infection in children.

Our study's main strength lies in the use of NIS 2020–2021 data, which provides a large sample size based on population sampling of hospitalized pediatric populations with respiratory viral infections. However, there are limitations to this approach. The NIS database utilizes hospital discharge records from all HCUP-participating hospitals, excluding rehabilitation and long-term acute care hospitals. It includes only inpatient records, so the findings may not be generalizable to outpatient settings or patients who were transferred between hospitals. The database relies on ICD-10 codes for diagnosing respiratory infections, which may lack detailed information on the procedures and tests used for diagnoses. Additionally, as with any medical and billing database, the NIS may contain incorrect or missing information, leading to potential inaccuracies. This study did not include cases with co-respiratory viral infections (e.g., COVID-19 + influenza, Influenza + RSV, etc.). Furthermore, we were unable to assess the influence of vaccination (RSV, COVID-19, Influenza) on clinical outcomes, as vaccination status is not captured in the database.

## Conclusions

In conclusion, our findings indicate that children with COVID-19 infections are at increased risk of cardiovascular complications, including myocarditis and bradyarrhythmia/heart block. We recommend measures to prevent COVID-19 infection and to anticipate and promptly manage these cardiovascular complications in risk-prone children especially those with underlying comorbid conditions to prevent mortality.

## Supplementary Information

Supplementary Material 1.

## Data Availability

The datasets generated and/or analysed during the current study are available on the HCUP website. https://hcup-us.ahrq.gov/.

## Abbreviations

COVID-19: Corona virus disease of 2019
RSV: Respiratory syncytial virus
RV: Rhino virus
SARS-CoV-2: Severe acute respiratory syndrome coronavirus 2
NIS: National inpatient sample
HCUP: Healthcare cost and utilization project
AHRQ: Agency for healthcare research and quality
ICD-10-CM: International classification of diseases, tenth revision, clinical modification
ECMO: Extracorporeal membrane oxygenation
IQR: Interquartile range
CI: Confidence interval
aOR: Adjusted Odds ratio
aHR: Adjusted Hazard’s ratio
ACE2: Angiotensin-converting enzyme 2
CDC: Centers for disease control and prevention
CHB: Complete heart block
