# Supplementary material for: Comparison of mortality and cardiovascular complications due to COVID-19, RSV, and influenza in hospitalized children and young adults
Source: BMC Cardiovasc Disord. 2024 Nov 28;24:686. doi: 10.1186/s12872-024-04366-0 (PMC11603966; doi:10.1186/s12872-024-04366-0)
Supplement: Supplementary file 1 — Supplementary Material 1. [file 12872_2024_4366_MOESM1_ESM.docx]

**Supplementary Table 1.** Variables and the corresponding ICD-10 codes.

| **Variables** | **ICD-10 code** |
| --- | --- |
| ***Procedures codes*** | |
| **Extracorporeal membrane oxygenation** | 5A1522F, 5A1522G, 5A1522H |
| ***Diagnostic codes*** | |
| **COVID-19** | U07.1, B97.29 |
| **Influenza** | J10.xx to J11.xx, J09X, J09X1,J09X2,J09X3, J09X9 |
| **RSV** | B97.4, J12.1, J20.5, J21.0 |
| **Congenital heart disease** | Q20.xx to Q26.xx |
| **Diabetes** | E08.xx to E13.xx |
| **Obesity** | E65, E66.xx |
| **Myocarditis** | I51.4, I40.8, I40.9, B33.20, I40.1, I40.0, J10.82, I41, B33.22, I40 |
| **Tachyarrhythmias** | I47.1, I47.2, I49.01, I49.0, I48.0, I48.3, I48.4 |
| **Bradyarrhythmia/Heart block** | I44.xx, I45.xx |
| **Sudden cardiac arrest** | I46.x |
| **Asthma/reactive airway disease** | J45.xx |
| **Prematurity (<37 weeks of gestation)** | P072.1 to P072.6, P073.x |
| **Chromosomal anomalies** | Q90.xx-Q99.xx |
